# Paternal depressive symptoms and factors associated among expectant fathers in a northeastern province of Thailand

**DOI:** 10.1038/s41598-024-65997-z

**Published:** 2024-07-01

**Authors:** Chattarika Loekdee, Pajaree Jungjamroonrat, Tanin Kongsila, Pranee C. Lundberg, Nitikorn Phoosuwan

**Affiliations:** 1 Faculty of Public Health, Kasetsart University, Chalermphrakiat Sakonnakhon Province Campus, 59/7 Moo 1 Chiangkrua, Muang Sakon Nakhon, Sakon Nakhon, 47000 Thailand; 2 Bungtawai Subdistrict Administrative Organization, Sakon Nakhon, Thailand; 3 Nong Muang Hospital, Lopburi, Thailand; 4 Faculty of Agriculture, Kasetsart University, Bangkok, Thailand; 5 Department of Public Health and Caring Sciences, Faculty of Medicine, Uppsala University, Box 564, 751 22 Husargatan 3Uppsala, Sweden

**Keywords:** Prevalence, Associated factors, Paternal depressive symptoms, Expectant fathers, Public health, Psychology

## Abstract

Globally, depression is a major mental health problem among expectant fathers. Therefore, factors associated with paternal depressive symptoms (PDS) need investigation. This hospital-based cross-sectional study was aimed to investigate the prevalence of and factors associated with PDS among expectant fathers in a northeastern province of Thailand. In the north-eastern province, Sakon Nakhon, 440 expectant fathers from eight hospitals participated in the study by completing a questionnaire related to socio-demographic characteristics, the Edinburgh Postnatal Depression Scale (EPDS), psychosocial factors and social support. An EPDS score of at least eleven out of 30 was interpreted as having PDS. Multivariable linear regression analysis was applied with a statistical significance at 0.05, and the coefficient β was presented. In total, 81 expectant fathers (18.4%, 95% confidence interval 14.6–22.3) had PDS, and the mean (standard deviation) of the EPDS score was 6.65 (4.25). Insufficient money (β = − 0.099, p = 0.016), marital adjustment (β = − 0.098, p = 0.027), self-esteem (β = − 0.150, p < 0.001), wife’s stress (β = 0.079, p = 0.049), and expectant father’s stress (β = 0.400, p < 0.001) were factors independently associated with PDS. In conclusion, screening expectant fathers during the pregnancy period of their wives is essential, and factors associated with PDS should not be neglected by healthcare providers. Also, there is need of an intervention program to prevent the symptoms, especially for expectant fathers having insufficient money or having stress.

## Introduction

Globally, depression is a major mental health problem. In particular, it may happen in the transition to fatherhood^1^. Due to their expanding roles associated with parenthood, men are especially anticipated to be at a status-defining age^2^. Depressive symptoms among expectant fathers (EFs) affect the way of living, working and acting as a father by taking care of wife and raising children^3^.

From a meta-analysis, the global prevalence of paternal depressive symptoms (PDS) during the pregnancy period was found to be 13.59% in the 1st, 11.31% in the 2nd and 10.12% in the 3rd trimester^4^. Prevalence of PDS was found to range between 3.3% at 12 and 4.1% at 36 weeks of gestational age among pregnant women in Asia^5^, and pregnant women having depressive symptoms may increase by seven times the chance of having depressive symptoms of their couples. Several factors are associated with PDS during the pregnancy period. They may be of personal, biological and psychosocial character^6^. Personal factors include age^7,8^, unemployment^9^, stress^10–12^, history of mental health illness^7,13^, child's gender^14,15^, low income^16^, and self-esteem^5^. Biological factors are related to the change of hormone levels in the body, and depression of fathers is associated with five types of hormonal changes: a decrease in estrogen levels, testosterone levels, cortisol levels, vasopressin levels, and prolactin levels^17^. Psychosocial factors related to PDS include social support from important people, from family and friends^8,13,18^, and from couple relationship^7,8,13,19^. In addition, family relationship, such as between mother-in-law and EFs, plays an important role among couples during the pregnancy period^20^.

In Thailand, only one antenatal study of PDS^21^ in a central province and two postnatal studies of PDS^18,19^ in a northern province have been carried out. In order to understand the situation of PDS and plan an intervention for a reduction of PDS, this study aimed to investigate the prevalence of and factors associated with PDS among expectant fathers in a northeastern province of Thailand.

## Methods

### Study setting

This cross-sectional study was carried out in Sakonnakhon, a north-eastern province of Thailand which is located 647 km from Bangkok with a population of approximately 1.2 million. It has eighteen government hospitals (one provincial hospital and seventeen community hospitals) with approximately 7000 childbirths each year^22^.

### Participants and procedures

A sample size calculation with Z_α/2_ = standard normal distribution curve critical value for 95% CI (1.96), Z_1/β_ = Testing power (0.8), β = the coefficient of X1 or log odds ratio is (0.80), and *P*_0_ = incident rate (0.214)^23^, results in sample size = 293. After consideration of attrition rate, the sample size was taken to be 440. Inclusion criteria were EFs (1) at least 18 years old, and (2) having a wife pregnant during 12–40 weeks. EFs who reported having mental health conditions (e.g. depression, dementia, and bipolar disorder) were excluded because they might not answer some sensitive questions in the questionnaire. Data were collected from July to October 2022 by use of a consecutive sampling method (collecting data among the EFs who came to the hospitals during the collecting time)^24^.

In total, 512 EFs with pregnant wives (12th to 40th week) were recruited from eight hospitals in Sakonnakhon according to the sizes of the hospitals (one provincial hospital and seven community hospitals). The directors of the selected hospitals approved the study plan and permitted data collection. A midwife retrieved a list of pregnant women and a trained research assistant invited partners of the pregnant women to participate in the study. The 440 out of 512 who agreed to participate (86% response rate) were requested to complete the questionnaire with help from the research assistant in a room of an ANC clinic where their wives received ANC service. Each EF self-answered the questionnaire in approximately 30 min and then returned it to the research assistant for check of its completeness.

### Ethical approval

Ethical approval was granted from ethics committees in Kasetsart University Chalermphrakiat Sakon Nakhon Province Campus (COA NO.COA65/010) and Sakon Nakhon Provincial Hospital (SKNHREC No. 006/2565). After permission from the director of the selected hospitals, this study was conducted in compliance with the ethical principles of the Declaration of Helsinki. All participants received both oral and written information before signing a consent form at the ANC clinic of each hospital. Informed consent was obtained from all participants.

### Measurements

This study used a structured questionnaire comprising six parts: (1) the socio-demographic backgrounds of the participants, (2) the Edinburgh Postnatal Depression Scale^25^, (3) the Rosenberg Self-Esteem Scale^26^, (4) the Dyadic Adjustment Scale^27^, (5) Stress assessment^28,29^, and (6) the Revised-Multi-dimensional Scale of Perceived Social Support (r-T-MSPSS)^30^. The first part was constructed by the researchers, and the other parts were constructed on the basis of literature review investigating factors associated with PDS^25–30^. The questionnaire had been tested for content validity by four experts with PhD qualifications using the Content Validity Index (CVI) in order to quantitatively determine the scale validity for the entire questionnaire (six parts). The CVI score was acceptable (Structure CVI = 0.98). The reliability test was conducted among 30 EFs in a province near Sakonnakhon, and the overall Cronbach’s alpha was found to be 0.70.

Part I: Personal factors (17 items) gathered information about age, education, employment, marital status, family type, self-determined income sufficiency, marital adjustment, and wives’ information from medical record (i.e. pregnancy history, anxiety, and stress).

Part II: The Edinburgh Postnatal Depression Scale (EPDS) is a depression screening scale developed by Cox and colleagues^31^. It consists of ten questions with four possible responses. Each item has a score of 0–3, and the total possible score ranged from 0 to 30 points. A Thai version of the EPDS^25^ was used with permission from Pitanupong and colleagues^25^ in which an EPDS score of 11 or more was defined as having PDS^21^. The reliability was tested and the Cronbach’s alpha coefficient was found to be 0.80.

Part III: The Rosenberg Self-Esteem Scale (RSES-Revised) was used to measure the father’s self-esteem. It had ten items, each with a score of 0–4, resulting in a total possible score of 10–40^32^. A Thai RSES-Revised was used with permission from Wongpakaran and Wongpakaran^26^. A score below 15 was considered to indicate low self-esteem. The Cronbach’s alpha for this part was found to be 0.82.

Part IV: The Dyadic Adjustment Scale (DAS) is a 14-item non-copyright questionnaire developed by Spanier^33^. It was translated into Thai by Phoosuwan, Eriksson and Lundberg (2018) in order to evaluate the marital relationship of couples^27^. The score ranged from 0 to 69 and a score of 48 or more indicated high marital adjustment while a score lower than 48 indicated low marital adjustment*.* This part had a Cronbach’s alpha 0.80.

Part V: Stress Test Questionnaire (ST-5) is a 5-item free-to-use questionnaire developed by the Department of Mental Health^28^ and Silpakit^29^, and each item could generate a score of 1–3. The total possible score range was 0–15. A score of 0–4 indicated having mild stress, 5–7 having moderate stress, 8–9 having high stress, and 10–15 having severe stress. The reliability test was confirmed, and the Cronbach’s alpha was found to be 0.84.

Part VI: Revised-Multi-dimensional Scale of Perceived Social Support (r-T-MSPSS) is a 12*-*item questionnaire developed by Zimet and colleagues^34^. There were 12 items, each of which can generate a score between one and seven, resulting in a total possible score of 12–84. A Thai version was used with permission from Wongpakaran and Wongpakaran^30^, in which a score of 12–36 meant having low perceived social support, 37–60 meant having moderate perceived social support and 61–84 meant having high perceived social support. The Cronbach’s alpha of this part was found to be 0.84.

### Data analysis

All data were analyzed using a Statistics Package for Social Sciences program. Descriptive statistics were used for socio-demographic data, such as mean and standard deviation (SD) and the variables were expressed as frequency and percentage (%).

Inferential statistics (multivariable linear regression analysis) was performed to explore the association between independent and dependent variables. The dependent variable was PDS (an EPDS score of 11 or more was classified as having depressive symptoms)^21^. The independent variables were (1) socio-demographic backgrounds of the participants such as age, group, educational level, employment and wives’ information, (2) self-Esteem, (3) marital adjustment, (4) stress and (5) perceived social support. The importance of each selected variable was verified following the fit of assumption model, containing the test of normality dependent variable (Z_skewness_ = 0.94, Z_Kurtosis_ = 1.98), the test of the multicollinearity problems, and the Variance Inflation Factor (VIF) respectively. In the univariable regression analysis, each independent variable was put into a model. The significant independent variables from the univariable regression analysis were retained in the multivariable regression analysis using an enter method. The R^2^ demonstrated percent that all significant independent variables were associated with PDS. All regression analyses used the significance level 0.05.

## Results

A total of 440 EFs participated in the study. The mean age and SD of the participants was 29.08 and 7.96 (range = 18 to 57 years). The majority of the participants had graduated from upper secondary school or equivalent. See Table 1.

**Table 1:** Characteristics of the participants (n = 440).

Characteristics	Frequency (%)
Age group (years)	
< 20	57 (13.0)
20–29	195 (44.3)
30–39	138 (31.4)
≥ 40	50 (11.3)
Mean ± SD (range) = 29.08 ± 7.96 (18–57)	
Educational level	
Primary	51 (11.6)
Lower secondary	90 (20.5)
Upper secondary or equivalent	156 (35.5)
Post-secondary	63 (14.2)
Tertiary	80 (18.2)
Employment	
Part-time	277 (63.0)
Full time	107 (24.3)
Unemployed	56 (12.7)
Marital status	
Married and living together	308 (70.0)
Not married but living together	115 (26.1)
Not married and living together	9 (2.0)
Married without living together	8 (1.9)
Income sufficiency	
Sufficient, no deposit	243 (55.2)
Sufficient, with deposit	140 (31.8)
Insufficient	57 (13.0)
Family type	
Extended family	286 (60.9)
Nuclear family	172 (39.1)
Administrative communities	
Local administrative organization	248 (56.4)
Municipality	192 (43.6)
Living together (years)	
1–5	340 (77.2)
6–10	83 (18.9)
≥ 11	17 (3.9)
Mean ± SD (range) of living together = 29.08 ± ± 7.96 (18–57)	
Religion	
Buddhism	421 (95.7)
Christianity	15 (3.3)
Muslim	2 (0.5)
No religion	2 (0.5)
Smoking behaviour	
No	156 (35.5)
Yes	172 (39.0)
Ex-	112 (25.5)
Alcohol consumption	
Yes	280 (63.6)
Ex-	105 (23.9)
No	55 (12.5)
Planned pregnancy	
Yes	313 (71.1)
No	127 (28.9)
Father's expectation on gender of the child	
Gender unknown	265 (60.2)
As expected	132 (30.0)
Not as expected	43 (9.8)
Health insurance (wife)	
Universal coverage scheme	305 (69.3)
Social security scheme	100 (22.7)
Government or state enterprise office	35 (8.0)
Current disease (wife)	
No	401 (91.1)
Yes (gestational diabetes mellitus/anemia)	39 (8.9)
Gravida status (wife)	
Primigravida	211 (48.0)
Multigravida	229 (52.0)
Parous status (wife)	
Nulliparous	231 (52.5)
Primiparous	141 (32.0)
Multiparous	68 (15.5)
Abortion history (wife)	
Never abortion	383 (87.0)
Abortion	57 (13.0)
Number of living of the child	
Never had children	383 (87.0)
1	57 (13.0)
2	383 (87.0)
≥ 3	57 (13.0)
Gestational age (wife)	
1st trimester	180 (49.9)
2nd trimester	151 (34.3)
3rd trimester	109 (24.8)

*SD* standard deviation.

The prevalence of PDS was 18.4% (95% CI = 14.6–22.3), where mean score and SD of the EPDS were 6.65 and 4.25, respectively. See Table 2.

**Table 2:** Prevalence and factors associated with paternal depressive symptoms of the participants (n = 440).

Characteristics	Frequency (%)
Prevalence (cut-off 10/11)	
No depressive symptoms	359 (81.6)
Having depressive symptoms	81 (18.4)
Mean ± SD (range) of the depressive symptom score = 6.65 ± 4.25 (0 – 22)	
Paternal depressive symptoms (n = 81)	
1st trimester of pregnancy	32 (39.5)
2nd trimester of pregnancy	31 (38.3)
3rd trimester of pregnancy	18 (22.2)
Stress (wife)	
Mild stress	172 (39.1)
Moderate stress	149 (33.9)
High stress	48 (10.9)
Severe stress	71 (16.1)
Mean ± SD (range) of the stress score = 5.62 ± 3.51 (0–15)	
Anxiety (wife)	
Low	397 (90.2)
Medium	29 (6.6)
High	14 (3.2)
Mean ± SD (range) of the Anxiety score = 3.58 ± 4.32 (0–21)	
Marital adjustment	
Low marital adjustment	176 (40.0)
High marital adjustment	264 (60.0)
Mean ± SD (range) of the marital adjustment score = 48.81 ± 9.29 (0–68)	
Self-esteem	
Low self-esteem	5 (1.1)
Moderate self-esteem	259 (58.9)
High self-esteem	176 (40.0)
Mean ± SD (range) of the self-esteem score = 29.98 ± 3.87 (10–38)	
Marital adjustment	
Low marital adjustment	148 (33.6)
High marital adjustment	292 (66.4)
Mean ± SD (range) of the marital adjustment score = 50.59 ± 8.27 (17–68)	
Stress	
Mild stress	279 (63.5)
Moderate stress	108 (24.5)
High stress	20 (4.5)
Severe stress	33 (7.5)
Mean ± SD (range) = 3.72 ± 3.05 (0–14)	
Social support	
Low	38 (8.6)
Medium	170 (38.6)
High	232 (52.8)
Mean ± SD (range) of the social support score = 59.39 ± 15.39 (12–84)	

*SD* standard deviation, *EPDS* Edinburgh Postnatal Depression Scale.

The findings revealed that insufficient income (β = − 0.099, p-value = 0.016), marital adjustment (β = 0.098, p-value = 0.027) and self-esteem (β = − 0.150, p-value = 0.001) were statistically significant factors associated with PDS. The dependent variables were able to predict 34.0% of factors associated with PDS (R^2^ = 0.34). See Table 3.

**Table 3:** Factors associated with paternal depressive symptoms by multivariable linear regression analysis (n = 440).

Variables	Unstandardized coefficients		Standardized coefficients	t	p-value	95% Confidence for interval for B	
	B	Std. error	Beta			Lower bound	Upper bound
Income sufficiency (EFs)	− 0.654	0.270	− 0.099	− 2.420	0.016*	− 1.185	− 0.123
Marital adjustment (EFs)	− 0.050	0.023	− 0.098	− 2.215	0.027*	− 0.095	− 0.006
Self-esteem (EFs)	− 0.164	0.047	− 0.150	− 3.499	< 0.001*	− 0.257	0.072
Stress (Wife)	0.095	0.048	0.079	1.978	0.049*	0.001	0.18
Stress (EFs)	0.556	0.059	0.400	9.489	< 0.001*	0.441	0.671
(Constant)	14.409	2.064	–	6.981	< 0.001	10.352	18.467
R = 0.58, R^2^ = 0.34, F = 22.77							

*Correlation was significant at the p-value < 0.05.

*EFs* expectant fathers.

## Discussion

The present study showed that the prevalence of Thai PDS during the pregnancy period was 18.4%. A study in a university hospital in the central region of Thailand found that PDS were 13.4% using the EPDS (EPDS ≥ 11)^21^, which is lower than found in the current study. The reason might be that the current study collected data among EFs living in rural areas. This is supported by Fellmeth and colleagues^34,35^ who found that living in a rural setting might lead to depression. In addition, Thai men are always considered as the breadwinners who support their family members^36^, and those in rural areas (especially in a northeast province in Thailand) might have had obstacles in life and fear of women’s health problems (e.g. gestational diabetes, experiencing pain during childbirth, or needing surgery) during the women’s pregnancy period^20^. These reasons might explain why the prevalence of PDS in this study is higher than in some other studies. Therefore, healthcare providers should identify EFs who are at risk of PDS, in order to give them relevant support, e.g. emotional support.

In this study, EFs having high marital adjustment might have reduced risk of having PDS. It has been found in several studies in Asia that low levels of marital relationship is strongly associated with PDS both before and after birth^5,37^. In particular, EFs during the gestation periods of 24–26 weeks had problems in their marriages^38^. Conflicts within families occur because EFs in Thailand are expected to live in their wife’s home with her family^20^. Low marital adjustment among EFs and their pregnant wives usually leads to depressive symptoms during pregnancy among women in the late antenatal period^27^. A parental intervention program for EFs and pregnant women can improve couple relationships, reduce depressive symptoms and prepare for their child^39^.

EFs may have increased level of stress during their wives’ pregnancy period. A high level of stress is related to mental health issues, such as depression and anxiety during the pregnancy period^13^ and also the postpartum period^40^. These stress problems result in school-age children with emotional and behavioral problems^41,42^, and a risk of physical abuse of the child^43^. The current study found that when the stress score increased, a number of depressive symptoms may increase. Not only pregnant women are screened for stress, the EFs also need to be considered for use of a screening tool, such as the EPDS, at least once during the pregnancy period^44^.

Most of the EFs in this study had insufficient income by their self-determination. Money insufficiency is a factor that affects depressive symptoms^16,45,46^. Sakon Nakhon is an agricultural region and a low-income province (Office of the National Economic and Social Development Council, 2022). The workers in Sakon Nakhon are unable to stop working because they get income on a day-by-day basis. Therefore, they need many working hours to maintain their income (Sakon Nakhon Provincial Labor Office City Hall, 2022). Currently, Thailand offers two different kinds of leave benefits for new fathers according to the government-specified guidelines: (1) government employees have a 15-day leave from work according to the regulation of the Office of the Prime Minister for civil servants, and (2) fathers have rights from the social security office to get a monthly allowance (600 Thai baht: 18 US dollars) for a child during the first six-year period. Due to income insufficiency and low support for new fathers, more support from government is recommended, such as a 60-day paternity leave and/or increased money allowance (e.g. 3000 Thai baht: 90 US dollars).

### Strengths and limitations

The strength of this study are: (1) the use of EPDS is globally validated to screen for depressive symptoms; (2) the EPDS score for PDS as a dependent variable was used to minimise misclassification of clinical diagnoses; (3) use of multivariable linear regression analysis could adjust for such confounders and demonstrate strengths of association; (4) this was a hospital-based study from government hospitals and most of EFs and pregnant women in Thailand use services from the government hospitals; and (5) a research assistant had been trained for data collection. Thus, the findings might be generalized to other EFs in similar contexts, e.g. those in a rural area or having low education. Lastly, this study had good quality of measurements.

There are also some limitations of this study. The tool used was a self-report rather than a medical record, some information might come more from participants’ thought than from evidence. Some factors, such as domestic violence and in-law family relationship were not investigated because they were not the main objective of investigation.

## Conclusion

The prevalence of PDS was 18.4% (95% CI 14.6–22.3). Insufficient money, marital adjustment, self-esteem, wife’s stress, and expectant father’s stress were factors independently associated with PDS. Screening EFs during the pregnancy period of their wives by healthcare staff using an instant tool is recommended, e.g. the EPDS. Those having insufficient money or having stress are population-at-risk for PDS and are in need of an intervention program.

## Data Availability

The datasets generated and/or analysed during the current study are not publicly available but are available from the corresponding author on reasonable request.
